# Contribution of NMDA Receptor Hypofunction in Prefrontal and Cortical Excitatory Neurons to Schizophrenia-Like Phenotypes

**DOI:** 10.1371/journal.pone.0061278

**Published:** 2013-04-16

**Authors:** Gregory R. Rompala, Veronika Zsiros, Shuqin Zhang, Stefan M. Kolata, Kazu Nakazawa

**Affiliations:** Unit on Genetics of Cognition and Behavior, National Institute of Mental Health, National Institutes of Health, Department of Health and Human Services, Bethesda, Maryland, United States of America; Rikagaku Kenkyūsho Brain Science Institute, Japan

## Abstract

Pharmacological and genetic studies support a role for NMDA receptor (NMDAR) hypofunction in the etiology of schizophrenia. We have previously demonstrated that NMDAR obligatory subunit 1 (GluN1) deletion in corticolimbic interneurons during early postnatal development is sufficient to confer schizophrenia-like phenotypes in mice. However, the consequence of NMDAR hypofunction in cortical excitatory neurons is not well delineated. Here, we characterize a conditional knockout mouse strain (CtxGluN1 KO mice), in which postnatal GluN1 deletion is largely confined to the excitatory neurons in layer II/III of the medial prefrontal cortex and sensory cortices, as evidenced by the lack of GluN1 mRNA expression in *in situ* hybridization immunocytochemistry as well as the lack of NMDA currents with *in vitro* recordings. Mutants were impaired in prepulse inhibition of the auditory startle reflex as well as object-based short-term memory. However, they did not exhibit impairments in additional hallmarks of schizophrenia-like phenotypes (*e.g.* spatial working memory, social behavior, saccharine preference, novelty and amphetamine-induced hyperlocomotion, and anxiety-related behavior). Furthermore, upon administration of the NMDA receptor antagonist, MK-801, there were no differences in locomotor activity versus controls. The mutant mice also showed negligible levels of reactive oxygen species production following chronic social isolation, and recording of miniature-EPSC/IPSCs from layer II/III excitatory neurons in medial prefrontal cortex suggested no alteration in GABAergic activity. All together, the mutant mice displayed cognitive deficits in the absence of additional behavioral or cellular phenotypes reflecting schizophrenia pathophysiology. Thus, NMDAR hypofunction in prefrontal and cortical excitatory neurons may recapitulate only a cognitive aspect of human schizophrenia symptoms.

## Introduction

NMDA receptor (NMDAR) hypofunction is one of the most prevalent models utilized in schizophrenia research. Healthy subjects treated with NMDAR antagonists, such as phencyclidine, ketamine and MK-801, show symptoms comparable to schizophrenic illness [1]–[4]. Furthermore, GluN1 (NR1) hypomorph mice, in which GluN1 expression is reduced to 5–10% of control levels, display deficits in social interaction and prepulse inhibition of the auditory startle reflex [5]. Though these findings implicate the NMDA receptor, little is known regarding which specific cell-types are crucial for the NMDAR hypofunction model of schizophrenia.

The cortex, a major target of schizophrenia pathophysiology [6], has two primary neuronal cell-types – glutamatergic excitatory neurons and GABAergic interneurons. Converging evidence has suggested that interneurons are particularly sensitive to NMDAR hypofunction [7]. For example, despite the abundant expression of NMDARs in excitatory neurons, acute systemic administration of NMDAR open-channel blockers results in hyperactivity of cortical pyramidal neurons [8], and spillover of cortical glutamate [9]. These findings suggest that constantly-depolarizing interneurons are preferentially affected by NMDAR hypofunction, resulting in net disinhibition of cortical excitatory neurons. Indeed, our lab has demonstrated that a postnatal deletion of NMDARs specifically in corticolimbic interneurons confers several behavioral and pathophysiological features in mice that resemble human schizophrenia [10].

Such evidence does not, however, negate the involvement of excitatory neurons in the NMDAR hypofunction model of schizophrenia. Indeed, numerous studies have implicated pyramidal neuron dysfunction in schizophrenia. Altered dendritic morphology and reduced spine density deficits have been repeatedly observed in the prefrontal cortex of postmortem schizophrenic patients [11]. NMDA receptors are critical to normal spine development in cortical pyramidal neurons *in vivo* [12]. Moreover, ablation of the schizophrenia risk gene dysbindin, which is highly expressed in excitatory neurons, yields lower NMDA currents in cortical pyramidal cells and impairs spatial working memory [13].

Although NMDAR deletion in cortical excitatory neurons has been reported in several conditional GluN1 knockout mouse lines, none have been suitable for conducting a thorough behavioral battery. The αCaMKII-Cre/GluN1 KO mutants [14] begin NMDAR deletion by postnatal 3–4 weeks and show Cre recombination throughout the entire forebrain [15]. Though exhibiting spatial reference memory deficits, this mutant line shows excessive hyperactivity which precludes an in depth analysis of psychiatric-like behavioral phenotypes. Other conditional GluN1 knockout lines, utilizing Emx1-Cre [16] and Nex-Cre [12], begin NMDAR deletion prenatally, and have significant weight reduction and lethality, respectively. Here we utilized a novel transgenic mouse line, G35-3-Cre/fGluN1, in which NMDARs are ablated postnatally in layers II/III of the prefrontal and cortical excitatory neurons, to delineate the extent to which NMDAR hypofunction in these neurons may contribute to schizophrenia-like phenotypes in adult mice.

## Results

### Spatial Distribution of Cre Recombinase in G35-3-Cre Line

**Figure 1:**
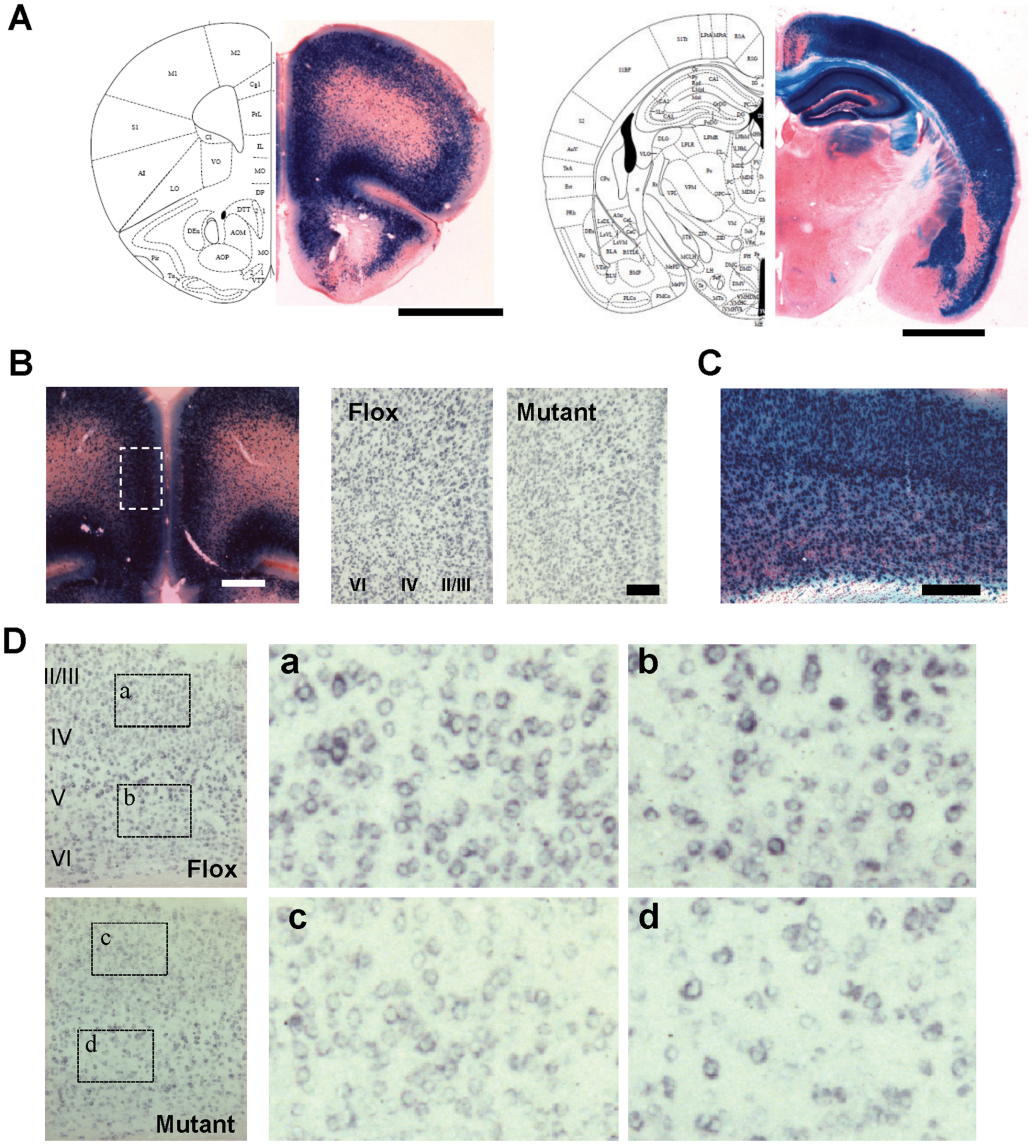
Histological characterization of cortical excitatory neuron-selective GluN1 knockout mice. **A.** Spatial distribution of Cre recombinase activity in coronal sections of G35-3-Cre/R26lacZ mice, stained with X-gal (blue) and Safranin-O. **B.** High magnification photographs in the medial prefrontal cortex (mPFC). Left, X-gal staining of G35-3-Cre/R26lacZ mice. Right, *in situ* hybridization images with DIG-labeled GluN1 cRNA (blue) in the mPFC region corresponding to the dotted area in the X-Gal staining image. **C** and **D.** High magnification photographs in the primary sensory (S1) cortex. X-Gal staining image in C, and *in situ* hybridization images with DIG-labeled GluN1 cRNA (blue) in D. Right panels, a to d, are shown from the dotted areas in the left panels from floxed-GluN1 control (Flox) and mutant mice, respectively. All mice were 11 weeks of age. Scale Bar: 200 µm.

We first characterized the Cre-recombination pattern of our G35-3-Cre line by crossing it with a *loxP-*flanked Rosa26LacZ reporter line. In offspring carrying the *lacZ* gene at 12 weeks of age, *lacZ-*positive neurons were largely confined to the majority of cortical areas, hippocampal proper and dentate gyrus, and amygdalal basolateral/lateral nucleus (Figs. 1A and S1). In the cortex, dense staining was detected in all prefrontal cortices (PFC) (including anterior cingulate, prelimbic, infralimbic, and orbitofrontal cortex), piriform cortex, motor cortex (only superficial layer), parahippocampal areas, and all the sensory cortices including somatosensory, auditory and visual cortex. However, neocortical areas close to the midline, such as posterior cingulate cortex, retrospleneal granular cortex, and primary motor cortex deep layer were largely spared (Fig. S1). Little or no *lacZ*-positive somata were detected in any other brain area, including olfactory bulb, dorsal striatum, nucleus accumbens, colliculus, thalamus, cerebellum, brainstem (Figs. 1A and S1), and spinal cord (data not shown). Previous histological characterization of this G35-3-Cre line revealed that the *lacZ* protein β-galactosidase does not co-label with Gad67-positive interneurons [17]. Therefore, Cre recombinase in G35-3-Cre mice was largely restricted to corticolimbic excitatory neurons.

### Cortical Excitatory Neuron-Targeted NMDAR Deletion

**Figure 2:**
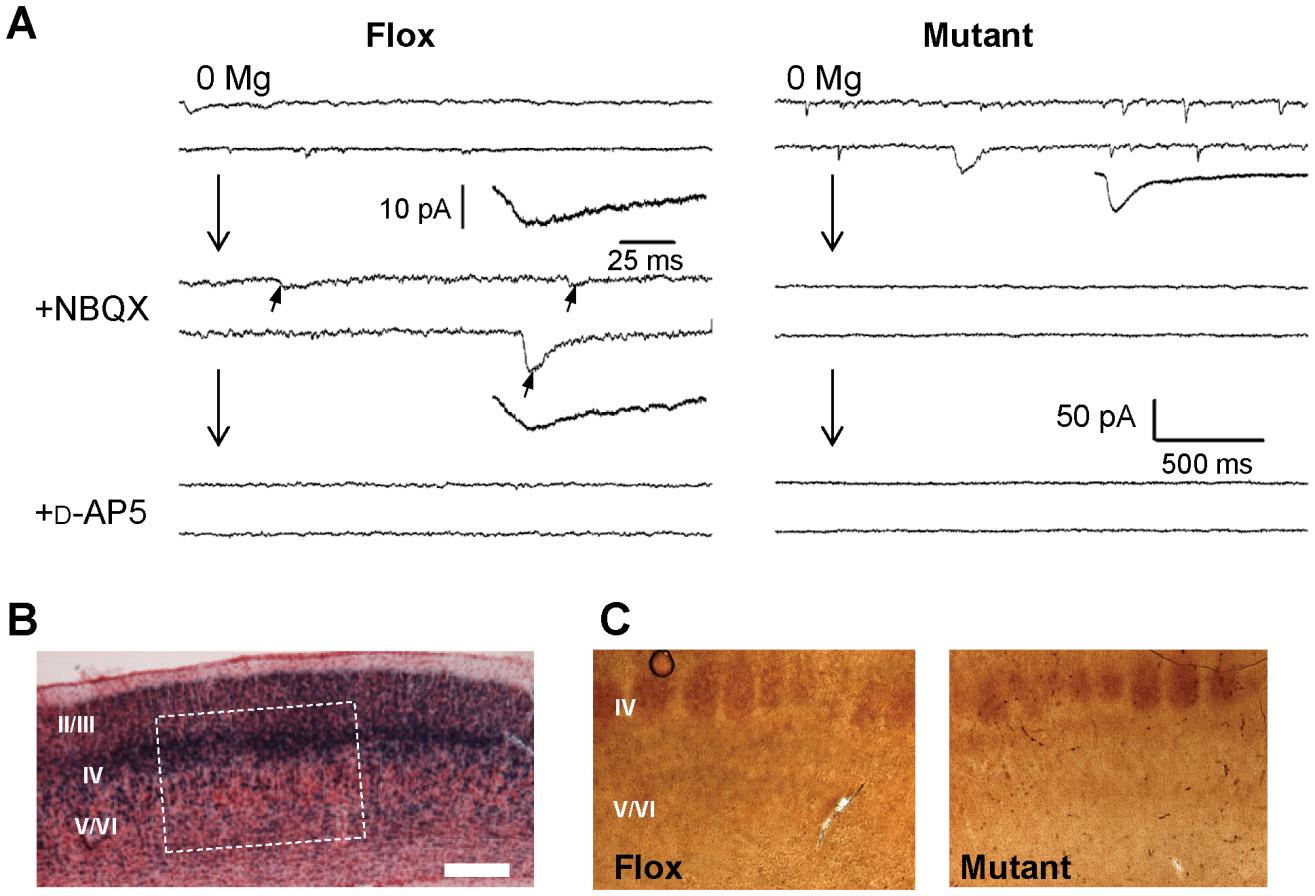
No NMDA currents detected in most of cortical layer II/III pyramidal neurons and postnatal onset of GluN1 deletion. **A.** An example trace (right) showing no NMDA currents were detected in mPFC layer II/III pyramidal neurons. Nine cells out of 10 cells tested from 7 mutants (12–17 weeks old) showed no NMDA currents, while they (arrows) were always detected in fGluN1 (Flox) controls. To isolate the NMDA component of spontaneous EPSC events, 20 µM NBQX was bath-applied, and, furthermore, 50 µM D-AP5 was added to ensure that NMDA channel currents were blocked. Scale bar is 500 ms and 50 pA for long traces and 25 ms and 10 pA for average excitatory currents. **B.** X-gal staining of the primary sensory (S1) cortex on postnatal day 6 (P6) of G35-3-Cre/R26lacZ mice. **C.** Immunohistochemistry staining for 5-HTT on P6 revealed no difference in barrel field development in the S1 cortex of fGluN1 (Flox) control and mutant mice. Scale bar: 200 µm.

To generate the cortical excitatory neuron-selective GluN1 KO (CtxGluN1 KO) mutant strain, we crossed the G35-3-Cre strain to a *loxP*-flanked GluN1 (fGluN1) strain [14]. Chromogenic *in situ* hybridization revealed that GluN1 mRNA signals are largely diminished from a majority of cells in both medial prefrontal cortex (mPFC; *i.e.,* anterior cingulate, prelimbic and infralimbic cortex) (Fig. 1B) and primary somatosensory (S1) cortex (Fig. 1C & D) at 10 weeks of age. To assess functional GluN1 deletion at a single-cell level, we performed whole-cell patch recordings of NMDA currents from visually identified mPFC layer II/III pyramidal neurons in 13–18 week old mice (Fig. 2A). In the Flox control mice, NMDA components of spontaneous EPSCs (sEPSCs) were detected in the presence of the AMPA channel blocker, 6-nitro-7-sulfamoylbenzo (f)quinoxaline-2,3-dione (NBQX), in all the cells tested (*n = *8), and their slower time-course sEPSCs (11.75±1.25 ms for 20–80% rise time; 19.04±2.76 ms for 66-30% decay time, see Table 1 for details) were similar to the values previously reported in the entorhinal cortex pyramidal neurons of adult rats [18]. These sEPSC events were subsequently blocked by the addition of the NMDA channel blocker *D*-(-)-2-Amino-5-phosphonopentanoic acid (D-AP5). In contrast, compared to the Flox controls, 9 out of 10 layer II/III pyramidal neurons in the mutants had sEPSC events with significantly shorter rise times and decay times in the Mg^2+^-free medium (Table 1), which were completely blocked by NBQX (Fig. 2A), suggesting these were AMPA components. Electrical properties of pyramidal cells were measured in some of these experiments and they were not significantly different between genotypes (Resting potential: −65.2±3.31 vs −66.4±2.13 mV, p = 0.79; membrane time constant: 25.6±5.95 vs 26.6±5.27 ms, p = 0.91 and input resistance: 234.5±25.1 vs. 211.4±30.0, p = 5.59, N = 5 and 5 for controls and mutants respectively. We also assessed the degree of GluN1 deletion in the mPFC deep layer. Nine out of 10 pyramidal neurons in the layer V/VI of the mutants had sEPSP events with long decay times, which were similar to the controls, suggesting these were NMDA components. As such, NMDA currents were detected in only 1 out of 10 pyramidal neurons in the mPFC layer II/III, whereas NMDA currents were present in 9 out of 10 pyramidal neurons in layer V/VI of the mutants. These results suggested that NMDA channel ablation is confined to layer II/III in the mPFC of mice.

**Table 1:** Characteristics of sEPSCs in mPFC layer II/III pyramidal neurons.

	Flox control (n = 8)		Mutant (n = 9)
	0 Mg^2+^	NBQX added	0 Mg^2+^
Peak amplitude, pA	14.94±1.51	11.75±1.29	13.79±1.60
20–80% Rise time, ms	7.50±1.15	11.25±1.75	3.17±0.77**
66-30% Decay time, ms	20.71±2.65	19.04±2.76	12.62±1.68*

* *p*<0.02 between genotypes.

** *p*<0.01 between genotypes. Data are mean ± s.e.m.

### Postnatal Onset of Cortical NMDAR Deletion

Cre recombination in S1 cortex layer IV excitatory neurons was already detectable at postnatal day 6 (P6) in G35-3-Cre/R26LacZ mice (Fig. 2B). To examine whether NMDAR deletion also occurs in the cortex at this stage, we assessed the degree of GluN1-dependent whisker-barrel formation in S1 layer IV [16]. Immunostaining for serotonin transporter (5-HTT), which is highly expressed by thalamocortical afferents in S1 cortex from postnatal day 5 to 9 [19], revealed no difference between fGluN1 control and CtxGluN1KO mice at P6 (Fig. 2C), suggesting that functional GluN1 deletion may be delayed until after postnatal 2nd week. Since mice with prenatal GluN1 deletion from corticolimbic pyramidal neurons do not survive past postnatal day 24 (P24) [12], postnatal onset of GluN1 deletion in those neurons may be critical for their survival.

### Mutant Mice Retain Hippocampal- and Amygdala-Dependent Behavior

**Figure 3:**
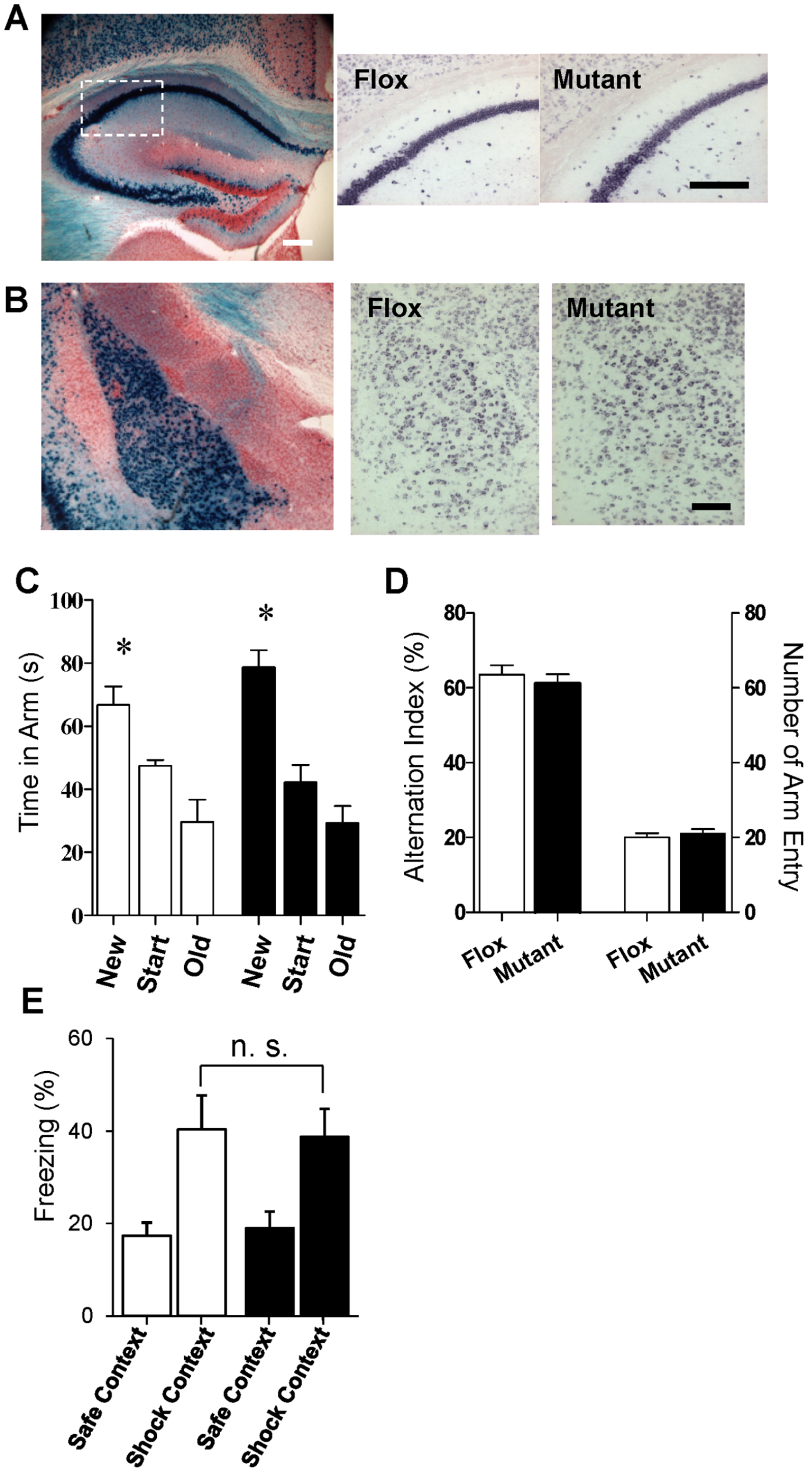
Integrity of hippocampus and amygdala. **A** and **B.** Eleven-week-old G35-3-Cre/R26lacZ mice showed robust Cre recombination (blue signals, left panel) in both hippocampal pyramidal neurons (A) and amygdalal principal neurons in lateral and basolateral amygdala nuclei (B). However, *in situ* hybridization revealed no clear reduction of GluN1 mRNA levels was detected in both brain regions of the mutant mice compared to Flox controls (right two panels) at the same age. In situ hybridization images corresponded to the white box area in the left panel. Scale Bar: 200 µm. **C.** In the spatial long-term memory task, both fGluN1 (while bar, n = 7) and mutant (black bar, n = 8) mice preferentially visited the novel arm during the test trial (two-way ANOVA/Fisher’s LSD *post-hoc* test: *p<0.05). **D.** In Y-maze spontaneous alternation task, no difference between fGluN1 (Flox, n = 17) and mutant (n = 16) mice in alteration percentage or arm entries. **E.** In the contextual fear conditioning test, there was no difference between the fGluN1 control (white bar, n = 9) and mutant (black bar, n = 10) mice for freezing in the training (Context A) versus the novel context (Context B) (repeated measures ANOVA; Group, *p* = 0.99; Group×Context, *p* = 0.78).

CtxGluN1KO mutant mice were viable into late adulthood, exhibiting normal body weight gain, motor coordination, and pain sensitivity (Fig. S2). Therefore, these mutants were ideal for use in a rigorous behavioral assessment. Although Cre recombinase activity was detected in the hippocampus and amygdala of G35-3-Cre/R26*lacZ* mice (Figs. 3A & B and S1), we found minimal difference between control fGluN1 and mutant mice in GluN1 mRNA expression in both regions (Fig. 3A & B). To assess the extent of functional NMDA channel deletion, we performed whole-cell patch recordings of NMDA currents from visually identified CA1 pyramidal neurons. We found that 6 out of 12 pyramidal neurons tested from 12 mutants showed NMDA component whereas the rest of the 6 neurons showed AMPA component only. To test whether such partial GluN1 deletions in these regions was sufficient to impair NMDAR-dependent behavior, we utilized spatial reference and working memory tests, which are hippocampus-dependent [20], as well as the contextual fear memory test, which is both hippocampus and amygdala dependent [21].

In the spatial reference memory Y-maze task (Fig. 3C), both fGluN1 and mutant mice preferred the novel arm to the old and start arms during the test trial 3 hr after initial training (two-way ANOVA, F(2,13) = 32.5, Fisher’s least significant difference (LSD) *post-hoc* test: *p*<0.05). There was no significant difference between fGluN1 and mutant mice for time spent in the new arm (LSD *post-hoc* test; *p* = 0.13). We also assessed mutant mice in the Y-maze spontaneous alternation task for spatial working memory (Fig. 3D). There was no difference between mutant mice and fGluN1 controls for arm entries (t(31) = 0.52, *p* = 0.61) or spontaneous alternation index (t(31) = 0.69, *p* = 0.50). In the contextual fear conditioning task (Fig. 3E), both fGluN1 control and KO mice froze significantly more to the shock-associated context A than to the control context B 24 hr after conditioning (repeated measures ANOVA within-subjects effect of context: F(1,17) = 15.87, *p*<0.01). There was no difference between genotypes for freezing to context A (effect of Group: F(1,17) = 0.00, *p* = 0.998, Group×Context: F(1,17) = 24.03, *p* = 0.786). The results of these three hippocampus-dependent tasks strongly suggest normal hippocampal function in mutant mice. Furthermore, mutants exhibited amygdala-dependent fear learning and memory. These behavioral experiments consistently showed functional integrity of both the hippocampus and amygdala in CtxGluN1 KO mice, suggesting that NMDAR hypofunction is largely restricted to the cortical areas including mPFC.

### Mutant Mice Exhibit Deficits in Cognitive Functioning

**Figure 4:**
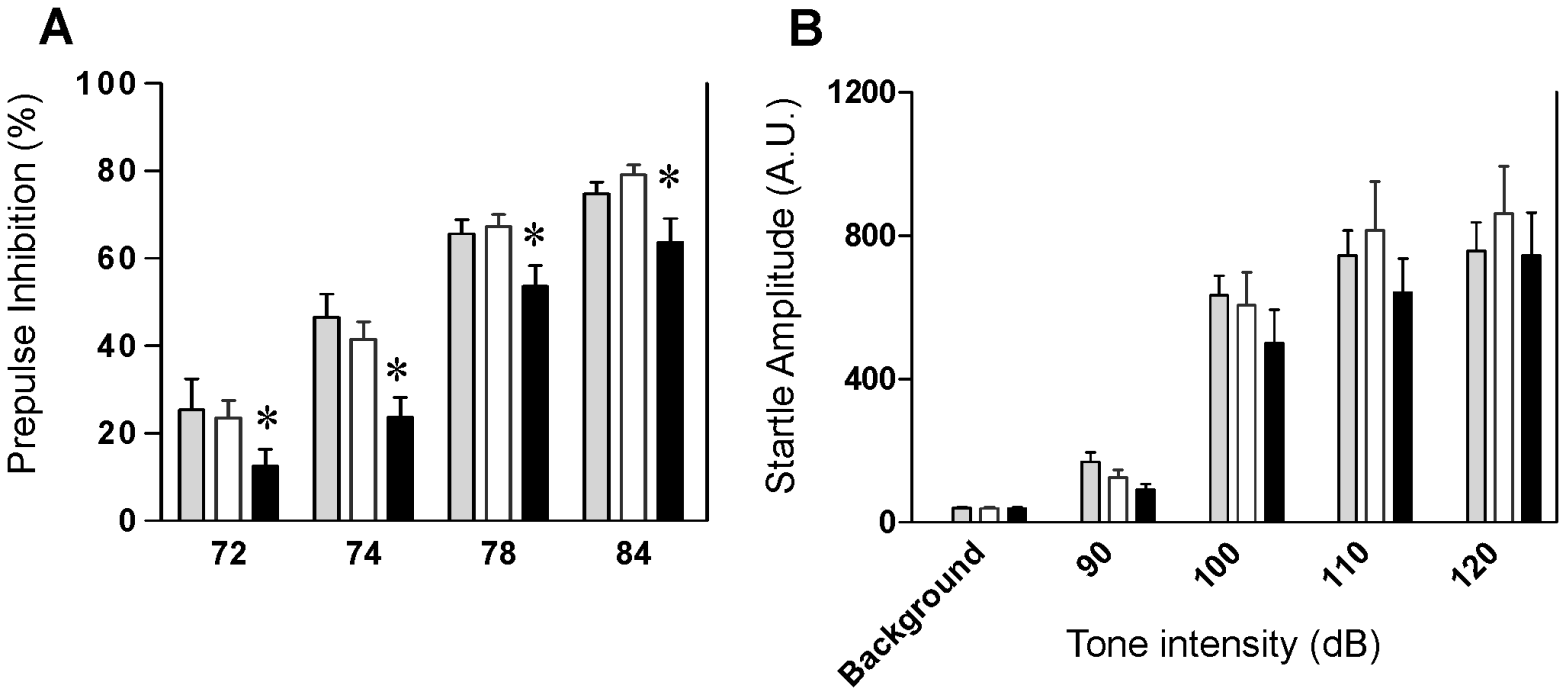
Mutants are impaired in prepulse inhibition of the auditory startle reflex. **A.** Compared with C57BL/6 wild-type (gray bar, n = 14) and fGluN1 (Flox) controls (while bar, n = 21), mutant mice (black bar, n = 20) showed significantly reduced prepulse inhibition of the auditory startle reflex (two-way repeated measures ANOVA; *p<0.05, for genotype factor). **B.** The same mutant mice (black bar) were unimpaired in auditory startle reflex compared to C57BL/6 wild type (gray bar) and Flox (white bar) controls (two-way repeated measures ANOVA; F(2,172) = 0.88, *p* = 0.42).

To test the validity of cortical excitatory neuron-targeted NMDA hypofunction as a model for schizophrenia pathophysiology, we subjected CxGluN1KO mutants and fGluN1 or wild-type control littermates to a battery of behavioral tests commonly used to measure schizophrenia-like phenotypes. Impaired prepulse inhibition of the auditory startle reflex is one of the most common phenotypes observed in genetic and pharmacological models of NMDAR hypofunction [5], [22]. Furthermore, PPI deficits in schizophrenia can be evoked by a deficit in attentional modulation of sensorimotor gating [23]–[24]. Compared with fGluN1 and wild-type controls, mutant mice showed significantly reduced PPI (Fig. 4A: two-way repeated measures ANOVA, F(2,162) = 5.97, *p*<0.0045). We also tested for auditory startle reflex as a control measure and found no difference between genotypes (Fig. 4B; two-way repeated measures ANOVA; F(2,172) = 0.88, *p* = 0.42). Since cortex-restricted GluN1 deletion is unlikely to impair brain stem-regulated sensorimotor gating, we interpreted the PPI deficit as a cortical deficit in attentional processing (see *Discussion*).

**Figure 5:**
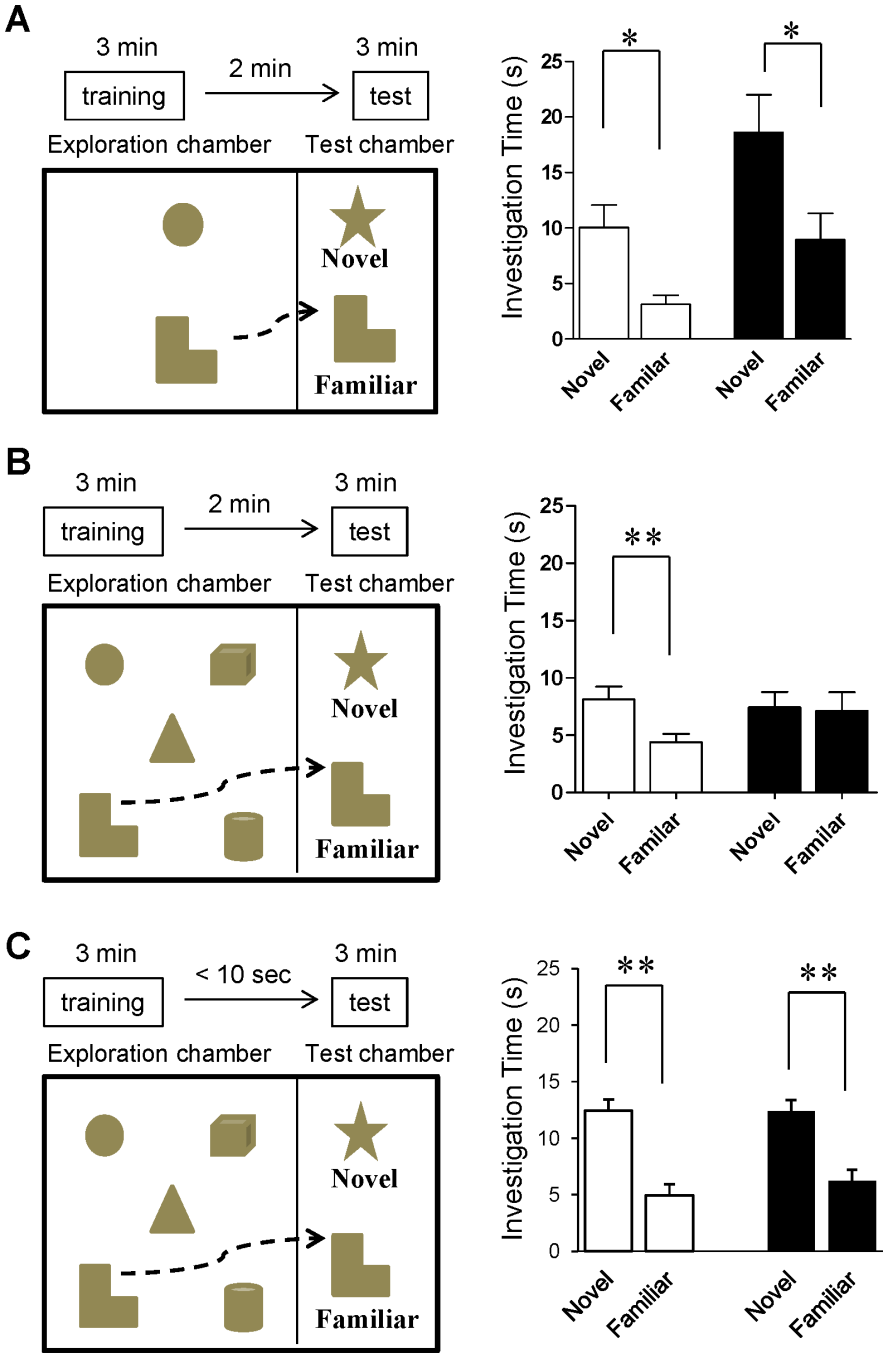
Mutants are impaired in multi-object short-term memory task. **A.** In a standard novel object recognition task with 2-min delay, both Flox controls (white bar, n = 7; two-tailed t-test, *p<0.05) and mutant mice (black bar, n = 8; two-tailed t-test, *p<0.05) spent more time investigating the novel vs old object. **B.** Two-min after a five-object exploration trial, Flox controls (while bar, n = 19) spent more time investigating the novel objects during the test trial (two-tailed t-test, **p<0.01), while mutant mice (black bar, n = 14) showed no difference for time spent investigating the novel vs old object (two-tailed t-test, *p* = 0.89). n.s., not significant. **C.** After a five-object exploration trial with almost no delay (<10 sec), both Flox controls (white bar, n = 8; two-tailed t-test, *p<0.05) and mutants mice (black bar, n = 9; two-tailed t-test, *p<0.05) spent more time investigating the novel vs old object.

Cortical NMDARs, especially in mPFC, have been hypothesized to play a role in cognitive functions such as working memory and attention [25]–[26]. Importantly, cognitive impairment is a core feature of schizophrenia [27]. To investigate mutant behavior in these cognitive domains, we adopted a novel object recognition task [28]. In this paradigm, mice are trained to recognize multiple objects in an open chamber and, after a short intertrial interval, are tested for object recognition with one novel and one familiar object from the training. By altering the delay period between training and testing and by altering the number of objects the animals are exposed to during training, the memory load can be adjusted. Critically, under high memory loads (2 min delay and 5 initial objects), we observed that the mutants, but not the controls, showed a deficit (Fig. 5B; t(13) = 0.14, *p* = 0.89 for mutant *vs* t(18) = 2.81, *p*<0.002 for Flox). To normalize object exploration time within subjects, we used the following equation to calculate a discrimination ratio for each animal: (novel object exploration time – familiar object exploration time/novel object exploration time+familiar object exploration time). Compared with fGluN1 controls, mutant mice were significantly impaired for discrimination ratio (0.32±0.08 for Flox, 0.040±0.087 for mutant, t(31) = 2.34, *p*<0.03). However, the mutant mice displayed significant novel object recognition when either the memory load was reduced to two objects (Fig. 5A; t(12) = 3.18, *p*<0.01 for Flox and t(14) = 2.33, *p*<0.05 for mutant) or when the post-training delay was less than 10 sec (Fig. 5C; t(14) = 4.31, *p*<0.001 for Flox and t(16) = 2.62 *p*<0.05 for mutant). Likewise, there was no significant difference in object discrimination ratio between genotypes for the two objects training condition (0.53±0.10 for Flox and 0.37±0.11 for mutant, t(13) = 1.05, *p* = 0.31) or for the five object and short delay condition (0.43±0.11 for Flox and 0.22±0.12 for mutant, t(15) = 0.96, *p* = 0.35). These results suggest that mutants are impaired in short-term memory capacity or in the attentional processes that are required for acquisition of a large memory load (see *Discussion*).

### Mutant Mice Displayed No Positive- or Negative-like Behavioral Deficits

**Figure 6:**
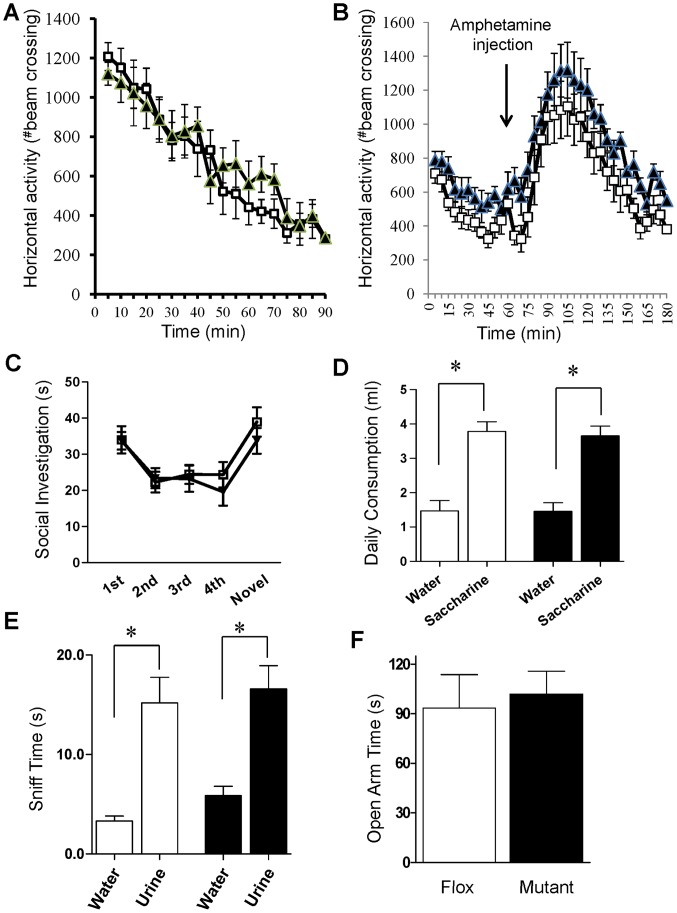
Positive and negative-like phenotypes. **A.** In the novel open field test, no difference in horizontal activity between mutant (black triangle, n = 8) mice and Flox (while square, n = 14) controls (two-way repeated measures ANOVA; *p* = 0.56). **B.** No significant difference for genotype (black for mutant (n = 9), white for Flox (n = 9)) in amphetamine-induced locomotor response (two-way repeated measures ANOVA after treatment; *p* = 0.12). **C.** No difference between mutant (black, n = 10) and Flox (white, n = 12) mice in the social recognition task (two-way repeated measures ANOVA; *p* = 0.56). **D.** In the saccharine preference test, both genotypes consumed saccharine more and no difference between mutant (black, n = 11) and Flox (white, n = 12) mice in saccharine preference (two-way ANOVA/LSD *post-hoc* test; *p* = 0.74). **E.** Mutant mice (black bar, n = 8) performed similarly to Flox controls (while bar, n = 9) in the female urine sniffing test (two-way ANOVA/LSD *post-hoc* test; *p* = 0.59). **F.** Mutant mice (n = 12) were comparable to flox controls (n = 11) in time spent in the open arm of the elevated plus maze (two-tailed t-test; *p* = 0.73).

Psychomotor agitation and psychostimulant sensitivity are two positive symptom-like behaviors commonly assessed in mouse models of schizophrenia [29]. In a novel open field chamber (Fig. 6A), no difference was observed in exploratory activity between fGluN1 controls and mutants (repeated measures ANOVA, F(1,15) = 1.6, *p* = 0.56). Mutant mice showed no difference in amphetamine-induced hyperlocomotion versus fGluN1 controls (Fig. 6B: repeated measures ANOVA after treatment, F(1,368) = 2.6, *p* = 0.12). Overall, we found no deficits implicating positive symptom-like deficits.

Schizophrenia is associated with negative symptoms such as impaired social interaction and recognition [30]. To examine the social behavior of mutant mice, we employed the social recognition task (Fig. 6C). There was no difference between mutants and fGluN1 mice in habituation to the demonstrator mouse (repeated measures ANOVA for first 4 trials, F(1,60) = 0.14, *p* = 0.72), initial investigation time towards the demonstrator mouse (LSD *post-hoc* test, *p* = 0.97 for first trial), or dishabituation when a new mouse was presented during the fifth trial (t(20) = 0.94, *p* = 0.36).

Another major negative symptom in schizophrenia is anhedonia which is most commonly modeled in mice using the saccharine preference test (Fig. 6D). Utilizing this test, we found a significant effect for saccharine vs water consumption (two-way ANOVA (F(1,42) = 63.58, *p*<0.001). There was no difference in saccharine consumption between mutant and fGluN1 control genotypes (LSD *post-hoc* test, *p* = 0.74). To further investigate pleasure-related phenotypes, we tested mutant mice for sexual pleasure-seeking deficits in the female urine sniffing test (Fig. 6E). Both mutant mice and fGluN1 controls showed a significant preference for urine vs water (two-way ANOVA (F(1,30) = 38.0; *p*<0.001). There was no difference in urine sniffing time for mutants vs fGluN1 controls (LSD *post-hoc* test, *p* = 0.59).

Finally, we tested CtxGluN1KO mice for naturalistic and anxiety-like behavior. In the home cage nesting test, there was no difference between genotypes as measured by weight of unused nestlet (0.15±0.15 g for mutant, 0.13±0.13 g for Flox, t(34) = 0.078, *p* = 0.94). In the elevated plus maze task (Fig. 6F), no difference was observed between fGluN1 control and mutant mice in both time spent in the open arm (t(21) = 0.35, *p* = 0.73) and number of open arm entries (6.9±1.2 for Flox, 9.4±1.5 for mutant, t(21) = 1.3, *p* = 0.21). Taken together, we did not detect any negative symptom-like behavior in the mutant mice.

### Mutant Mice Exhibit Normal MK-801-induced Hyperlocomotion

**Figure 7:**
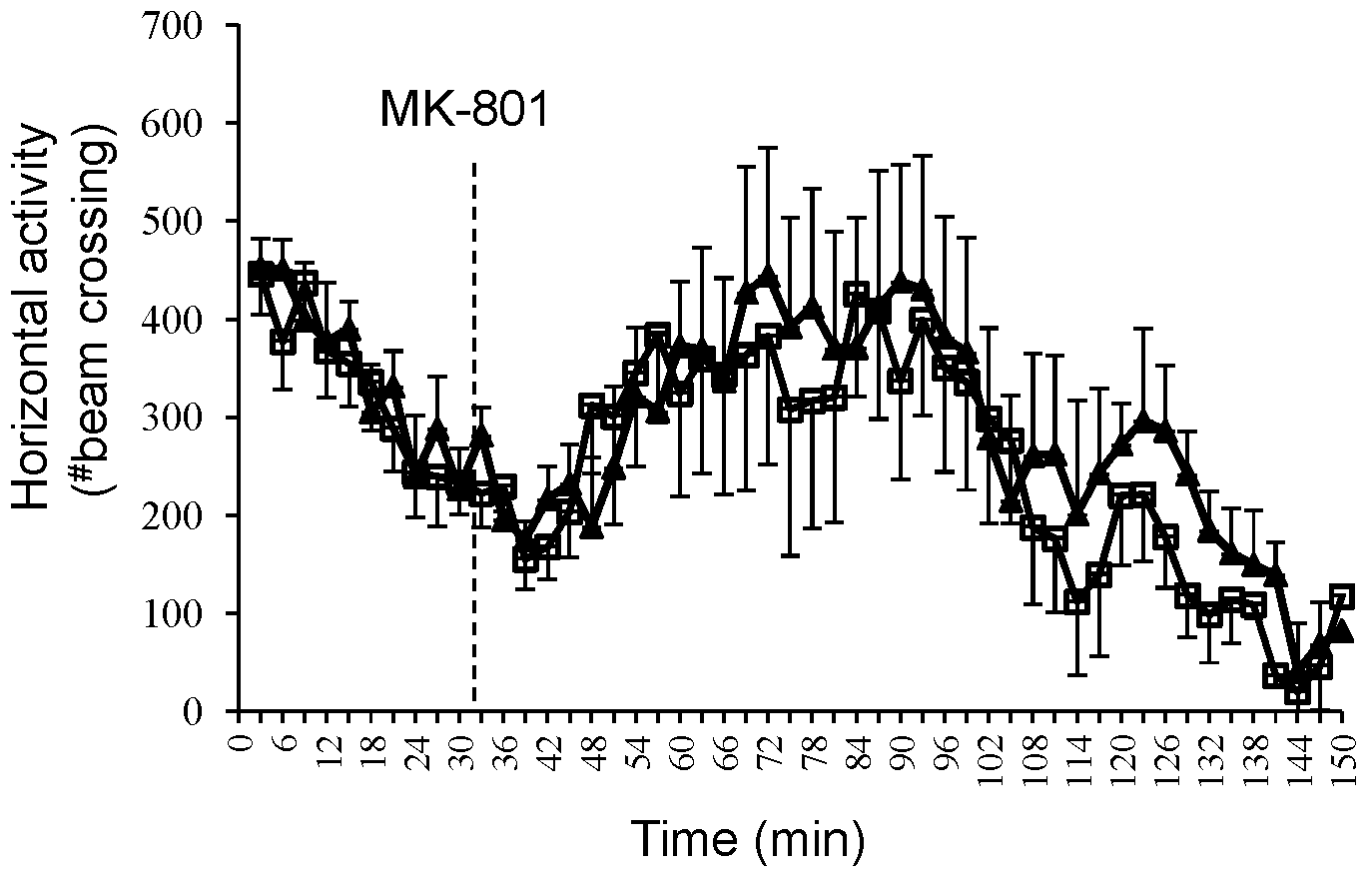
Normal MK-801-induced hyperlocomotion. No difference for genotype (black for mutant (n = 8), white for Flox mice (n = 9)) in MK-801-induced locomotor response (two-way repeated measures ANOVA after treatment; *p* = 0.72).

In rats and mice, moderate doses of the NMDAR antagonist MK-801 induce locomotor hyperactivity, which may reflect psychotomimetic action of NMDAR antagonists [29]. However, MK-801 was found to be ineffective in GluN1 hypomorph mice [5] and in the interneuron-specific NMDAR deletion mice [10], [31]. Therefore, if NMDAR hypofunction in cortical excitatory neurons is associated with MK-801-induced locomotor activity, CtxGluN1 KO mutants should exhibit altered response to MK-801. Remarkably, there was no difference in treatment-induced locomotor hyperactivity between fGluN1 control and mutant mice (Fig. 7: two-way repeated measures ANOVA after treatment (F(1,585) = 0.13, *p* = 0.72), suggesting that NMDAR hypofunction at cortical excitatory neurons is not attributable to MK-801-induced hyperlocomotion.

### Mutant Mice were Unimpaired in Oxidative Stress Response and GABAergic Inhibition

**Figure 8:**
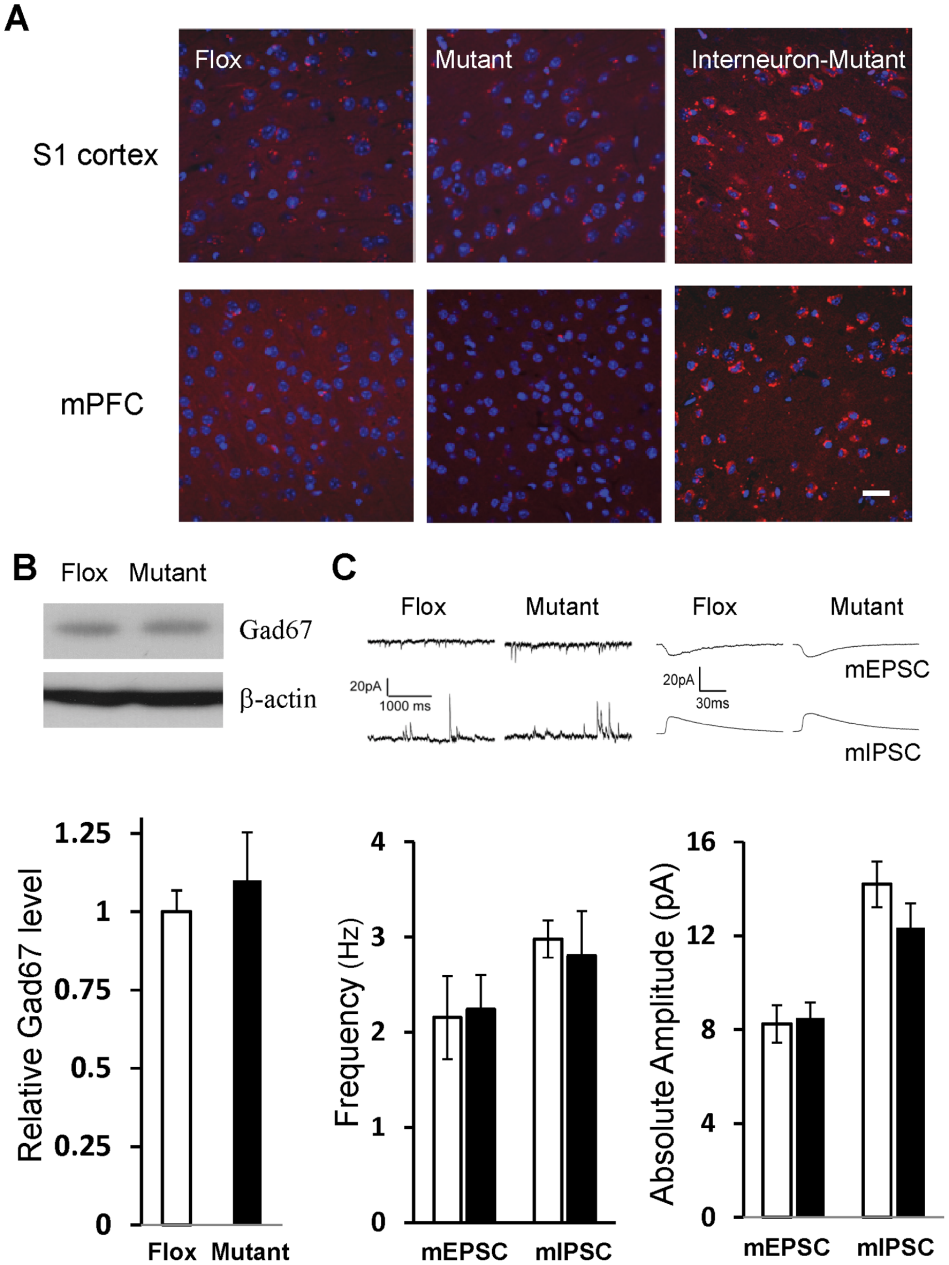
Reactive oxygen species’ level and GABAergic inhibition. **A.** Fourteen-week-old mice received injection of dihydroethidium to assess reactive oxygen species (ROS) levels. ROS levels, visualized by red fluorescence, were negligible in cortical sections of somatosensory cortex (upper) and medial prefrontal cortex (lower) in both Flox and CtxGluN1KO mutant mice. Right panels are sections from adult corticolimbic interneuron-GluN1 knockout mice (Ppp1r2-Cre/GluN1 KO mice) as a positive control. Scale Bar: 100 µm. **B.** Gad67 protein levels in cortical homogenates (6 animals for each group) were examined by Western blot. Gad67-IR intensity was normalized by the β-actin–IR on the same gel. No genotypic difference was detected in Gad67 protein levels. Student’s t-test, *p* = 0.56. **C.** Properties of miniature EPSC (mEPSC) and miniature-IPSC (mIPSC) events in mPFC layer II/III pyramidal neurons. The data of 2–3 cells were collected from single animal and averaged per animal (animal number, n = 6 for Flox (white bar), n = 7 for mutant (black bar)). No genotypic differences were observed in frequencies or amplitudes of both measures. Student’s t-test, *p* = 0.88 for mEPSC frequency, *p* = 0.97 for mIPSC frequency, *p* = 0.81 for mEPSC amplitude, *p* = 0.37 for mIPSC amplitude.

Converging lines of evidence suggest that the antioxidant defense system is compromised in schizophrenic patients [32]. Indeed, Ppp1r2-positive interneuron-specific deletion of NMDARs results in a robust increase of reactive oxygen species (ROS) production in S1 cortex and mPFC *in vivo* at 14 weeks of age (Fig. 8A; also see [33]). In comparison, CtxGluN1KO mutant mice show no significant increase in basal levels of ROS in S1 cortex and mPFC. Next, we assessed the cortical glutamic acid decarboxylase-67 (GAD67) protein level, a reduction of which is one of the most consistent findings in postmortem schizophrenic brains [34]. First, using western blot analysis, we found no difference in GAD67 protein level in the cortex between mutant and fGluN1 control mice (Fig. 8B). To further explore whether cortical GABAergic inhibition is dysregulated following postnatal GluN1 deletion from excitatory neurons, we conducted single cell-recordings from mPFC layer II/IIII pyramidal neurons in mutant slices at 10–15 weeks of age. No significant differences in the amplitude or frequency of both miniature excitatory postsynaptic current (mini-EPSC) and miniature inhibitory postsynaptic current (mini-IPSC) events were detected between the genotypes (Fig. 8C). Interestingly, our findings in mini-EPSC measurement were somewhat different from those obtained from prenatal GluN1 deletion mutants, which showed an increase in both amplitude and frequency of mini-EPSCs [12], possibly due to the different onset of GluN1 deletion or the age of data collection. In summary, these results suggested that postnatal NMDAR deletion from cortical pyramidal neurons has little impact on local GABAergic network function.

## Discussion

In the present study, we used behavioral, cellular, and physiological assays to characterize the downstream effects of cortical excitatory neuron-selective NMDAR deletion in mice. While the neocortex was not entirely affected, NMDARs were eliminated from layer II/III excitatory neurons in the mPFC and most sensory cortices during the postnatal period (Fig. 1; also see [17]). This restricted deletion yielded cognitive deficits in PPI and object-based attention tasks, but did not manifest in any positive or negative symptom-like phenotypes. In addition, mutants showed no obvious cellular deficits in oxidative stress regulation or cortical inhibitory tone. These results stand in stark contrast to the robust expression of schizophrenia-like phenotypes observed in the cortical and hippocampal interneuron-selective NMDAR knockout (Ppp1r2-Cre/fGluN1 KO) mutant mice [10],[33]. These robust differences between cell type-selective NMDAR knockout mice advance our understanding of NMDAR hypofunction in schizophrenia pathophysiology [35].

While excitatory cell-selective expression of Cre recombinase in the G35-3-Cre line was previously characterized [17], the brain areas where Cre-mediated recombination occurred were carefully re-examined by X-Gal staining. Consistent with the prior report, Cre recombination was largely confined to the mPFC and a majority of cortical areas, including somatosensory, auditory and visual cortices. However, since GluN1 deletion requires Cre recombination at both alleles of chromosomes, frequency of NMDAR deletion in CxGluN1 KO mutants may be more restricted compared to *lacZ* expression, which is only dependent on mono-allelic recombination. Although *in situ* hybridization for GluN1 mRNA revealed substantial GluN1 deletion in the S1 cortex and mPFC, it was unclear whether GluN1 gene was deleted at the both alleles or only at the single allele. NMDA current measurement revealed that layer II/III pyramidal neurons are largely devoid of NMDA currents, whereas NMDA channels are present in layer V/VI pyramidal neurons of the mPFC. Albeit not conclusive, together with previous finding showing that NMDA currents are undetectable in a majority of primary visual cortex in G35-3-Cre/fGluN1 mutants [17], these results suggested NMDAR deletion takes place in the mPFC layer II/III pyramidal neurons, and perhaps also in sensory cortices.

*In situ* hybridization data demonstrated a slight reduction of mutant GluN1 mRNA signal versus Flox controls in the hippocampal CA1 pyramidal neurons (Fig. 3A) while showing no detectable difference in the amygdala (Fig. 3B). However, mutants were unimpaired in the fear memory recall following contextual fear conditioning (Fig. 3E), which is known to be dependent on the integrity of both the hippocampus and amygdala [21]. Furthermore, spatial memory, both long-term and short-term, was also unimpaired (Fig. 3C & D). Notably, the Y-maze spontaneous alternation task for spatial working memory depends on the hippocampus, but not the mPFC in rodents [36]–[37]. Selective hippocampal manipulations, such as dorsal CA1 selective excitotoxic lesion [20], NMDAR deletion from dentate granule cells [38] and disruption of CA1 interneuron-mediated inhibition [39], result in spatial working memory deficits, supporting a critical role for rodent hippocampal function in spatial working memory. Therefore, while single cell-NMDA current measurement revealed that ∼50% of CA1 pyramidal neurons are devoid of NMDA channels, the remaining of 50% population of CA1 pyramidal cells may be sufficient to support functional integrity of hippocampus in the mutants.

Attentional function is severely disrupted in schizophrenia [40]. PPI is generally recognized as a simple operational measure of sensorimotor gating [41] and the primary circuitry mediating PPI resides in the brainstem [42]–[43]. However, as GluN1 deletion was minimally targeted to subcortical areas in the mutants, the most parsimonious explanation for the observed PPI deficit (Fig. 4A) could be impaired top-down attentional modulation of PPI from the cortex. For example, one study examined PPI in both the active and passive attentional paradigms in schizophrenic subjects and found that subjects showed less PPI particularly in the active attention phase [44]. Filion and Poje [23] further argued that schizophrenia-linked deficits in PPI are specific to the attentional modulation of sensorimotor gating, with no evidence of impaired automatic sensorimotor gating *per se*. Conversely, animal data suggest that a prepulse stimulus that has strong salience results in increased PPI [45]–[46]. PPI is not solely an automatic gating process, but includes top-down attentional components [47]. Therefore, we speculate that the PPI deficit observed in our mutant mice is due to impaired attentional modulation. Indeed, microinfusions of NMDAR antagonists into mPFC in rats have been shown to induce attentional impairments and increase impulsivity [48]–[50]. Interestingly, GluN1 hypomorph mice display deficits in PPI [51]–[53] as well as impaired selective attention, as measured by auditory event-related potentials [53]. It is plausible that the reported attentional impairment in GluN1 hypomorph mice could reflect NMDAR hypofunction in cortical excitatory neurons.

In addition to PPI, CtxGluN1KO mice appeared to exhibit a reduced object-based short-term memory capacity (Fig. 5). The phenotype was only evident when the short-term memory load was high (five objects to remember) but was absent under less demanding conditions (two objects to remember or very short delays). Many studies with rodents have characterized the role of NMDARs in working memory [54]. In one study, rats treated with the competitive NMDA receptor antagonist AP5 were impaired in a delayed matching-to-place version of spatial working memory [55]. It is noted that operational definitions of working memory for rodents which typically requires that unique stimuli be presented during a single learning trial, are different from those applied in human research, in which working memory is defined as a short-term memory capacity requiring conscious rehearsal and/or attention. However, MacQueen et al. [56] recently demonstrated that rats treated with MK-801 are impaired in an olfactory memory span task in which recall of an increasing number of olfactory stimuli was required as the session progressed. While this task may not be a direct measure of working memory capacity, the result supports the interpretation in this study that CtxGluN1 KO mutants are impaired in short-term memory when memory load is higher. Alternatively, albeit not mutually exclusive, it is also plausible that an attentional deficit affected the mutants during the exploration period, contributing to the recognition impairment [28], an interpretation consistent with the attentional deficits observed in the PPI task.

Aside from cognitive impairments, CtxGluN1 KO mutants performed comparably to fGluN1 controls in other tasks that are closely linked to schizophrenia-like phenotypes. For example, augmented amphetamine-induced locomotor hyperactivity has been used as a measure of altered dopaminergic neurotransmission in the mesolimbic system and has construct validity for enhanced dopaminergic activity in schizophrenia [31]. However, CtxGluN1 KO mice responded normally to amphetamine treatment, which is in sharp contrast to the excessive hyperlocomotion phenotype seen in corticolimbic interneuron-targeted Ppp1r2-Cre/fNR1 KO mice (Paredes *et al.,* manuscript in preparation). The CtxGluN1 KO mutant mice also lack other negative symptom-like deficits in female urine sniffing, and anxiety-like behavior. Therefore, the behavioral phenotypes observed in CtxGluN1KO mice may be dissociable from schizophrenia-like symptoms. Supporting this interpretation, CtxGluN1 KO mice failed to model two commonly observed cellular deficits in schizophrenic patients, increased oxidative stress markers [32] and reduced GAD67 expression [57]–[59], both present in Ppp1r2-Cre/GluN1KO mutant mice.

In summary, postnatal NMDAR elimination from prefrontal and cortical sensory excitatory neurons resulted in a cluster of cognitive dysfunctions related to attentional modulation and/or short-term memory. While similar deficits in PPI and spatial working memory were observed in mutant mice with postnatal NMDAR deletion in corticolimbic interneurons [10], the underlying mechanisms could differ, which may offer distinct therapeutic targets. More important, aside from cognitive impairments, cortical excitatory neuron-restricted GluN1 deletion did not manifest in any additional behavioral or cellular schizophrenia-like phenotypes (*e.g.,* positive and negative symptom-like behaviors, super-sensitivity to psychostimulant and social stress, and GABAergic dysfunction). This stands in contrast to interneuron specific NMDAR KO mice in which all of these deficits were observed [10], [33]. It is plausible that a GluN1 deletion largely restricted to the excitatory neurons of layer II/III mPFC and sensory cortices could obscure the more robust phenotypes in our mouse model. However, we observed no alteration in local synaptic inputs to the GluN1-deleted mPFC pyramidal neurons (Fig. 8C), suggesting that GluN1-deletion from cortical pyramidal neurons is unlikely to elicit cortical GABAergic dysfunction that may be crucial for schizophrenia pathophysiology [59]. Further studies are warranted to clarify the impact of NMDAR hypofunction in cortical excitatory neurons on inhibitory network function. Taken together, our findings suggest that NMDAR hypofunction in prefrontal and cortical sensory excitatory neurons may have preferential contribution to a cognitive domain of schizophrenia-like phenotypes. Among cortical neuronal cell-types, NMDAR hypofunction in the interneurons, rather than excitatory neurons, appears to trigger more variety of schizophrenia-like phenotypes [35].

## Materials and Methods

All experiments were carried out in strict accordance with the Guide for the Care and Use of Laboratory Animals of the National Institutes of Health. The protocol was approved by the National Institute of Mental Health Animal Care and Use Committee (Protocol Number: UGC-01-11).

### Animals

The generation of G35-3-Cre line was previously described [17]. In brief, a bacterial artificial chromosome (BAC) DNA fragment (∼120-kb long) containing genomic DNA sequence of the putative promoter, exon 1, and exon 2 of the glutamate receptor gene GRIK4 (KA-1) was co-injected into fertilized C57BL/6 mouse eggs with a DNA construct carrying both the minimal heat shock promoter and the Cre cDNA with nuclear localization signal (nls). Out of eight founder lines, in which both nls-Cre cDNA and BAC DNA sequences were co-integrated into the genome, was referred to as G35-3-Cre line. In the present study, in order to restrict the NMDAR deletion to the cortical excitatory neurons, we generated a novel G35-3-Cre^(+/−)^/homozygously-floxed GluN1^(loxP/loxP)^ knockout (CtxGluN1 KO or simply mutant) mice as follows. After initial crossing of hemizygous G35-3-Cre^(+/−)^ mice to a *loxP*-flanked GluN1 mice [14], offspring hemizygous for Cre transgene and heterozygous for fGluN1 allele was crossed to the mice homozygous for fGluN1 alleles to generate GluN1 KO animals with an expected frequency of 25%. The female KO mice were further crossed to homozygous-fGluN1 males to produce next-generation mutants and control fGluN1 littermate mice. Homozygous fGluN1 and C57BL/6 mice were used as controls. To characterize the spatial and temporal pattern of Cre recombination in G35-3-Cre mice, we crossed this line with a homozygous Rosa26-*lacZ* reporter line (B6;129S4-Gt(ROSA)26Sor/J) and stained the brain sections of progeny with X-Gal as previously described [10].

### Immunocytochemistry

Serotonin transporter (5-HTT/SLC6A4) staining of S1 cortex barrel fields at postnatal day 6 was performed as previously described [60]. Briefly, 50-µm thick coronal sections were cut by vibratome, washed in 1×PBS, blocked 1 hr in 10% normal goat serum, and incubated with anti-5HTT (1∶1000, Calbiochem) overnight. Sections were then incubated for 2 hr with biotinylated secondary antibody (1∶200), followed by ABC solution and 3,3′-Diaminobenzidine staining for chromogenic visualization.

### Non-radioisotopic In Situ Hybridization

The complementary RNA (cRNA) probe to mouse *GluN1* mRNA was derived from the AvrII-SphI 0.4 kb antisense DNA fragment of rat *GluN1* cDNA from exon 13 to exon 16 and labeled with digoxygenin (DIG) [61]. We postfixed 18-µm fresh frozen brain sections from 11-week-old mice for 15 min in 4% PFA-PBS, permeabilized them in 5 µg ml^−1^ proteinase K and acetylated them in 0.25% acetic anhydride (vol/vol) in 10 mM triethanolamine (pH 8.0). DIG-labeled *GluN1* mRNA antisense probe was hybridized at 56°C for 48 hr. After washing with graded SSC buffers, the DIG probe was incubated with sheep alkaline phosphatase–conjugated antibody to DIG (1∶2000, Roche) and DIG signals were detected using NBT-BCIP (blue).

### Western Blot

Whole cortex samples were dissected out and homogenized and analyzed by Western blot as previously described [10] using monoclonal anti-Gad67 (1∶5000, MAB5406, Millipore) and mouse anti-β-actin (1∶10000, A2228, Sigma-Aldrich). Western blot results were quantified using KODAK Molecular Imaging Software (Carestream Health Molecular Imaging, New Haven, CT).

### In Vivo Detection of Reactive Oxygen Species Level

*In vivo* detection of reactive oxygen species (ROS) in mouse brains was performed as previously described [62]. Briefly, two serial *i.p*. injections of freshly prepared dihydroethidium (DHE, 27 mg/kg, Invitrogen) were given at 30 min intervals. Eighteen hr later, mice were perfused with 4% paraformaldehyde in PBS. Brains were coronally sectioned on a vibratome with 35-µm thickness and counterstained with DAPI (4′, 6-diamidino-2-phenylindole). After mounting with Vectashield (Vector Laboratories; Burlingame, CA), 6 sections from each animals were observed under a confocal microscope.

### Slice Physiology

Whole-cell recordings were made from mPFC pyramidal neurons and hippocampal CA1 pyramidal neurons of the mutant mice and from their fGluN1 control littermates (12–18 weeks old) to evaluate the presence of NMDA currents and properties of inhibition and excitation. Miniature events were collected in 400-µm coronal slices (for mPFC measurements) or transversal (for hippocampal measurements) using conventional whole cell recording techniques. Intracellular solution contained (in mM): 125 Cesium-methane-sulfonate, 4 ATP-Mg^2+^, 4 NaCl, 0.3 GTP, 16 KHCO_3_, 5 N-2(2,6-dimethylphenylcarbamoylmethyl)triethylammonium chloride (QX-314-Cl), and equilibrated with 95% O_2_ and 5% CO_2_ to pH 7.3. The final osmolarity was 270–290 mOsm. The liquid junction potential was subtracted off-line and included in all the holding potential values throughout the paper. Recording temperature was set to 32–34°C.

Miniature excitatory postsynaptic events were collected in voltage clamp mode in the presence of TTX at a concentration of 500 nM at a holding potential value of −67 mV, while inhibitory postsynaptic events were collected at +3 mV as outward events in the same experiments. Miniature events were first collected in 60-s long traces using the template-based analysis feature of Clampfit 10.0. Events with peak amplitude values at least of 2-fold the standard deviation were included in the sample population.

We also recorded NMDA currents, as previously described [10] with some modifications. In brief, ACSF contained 12.5 µM gabazine (SR-95531; GABAA receptor antagonist), 1 µM (2S)-3-[[(1S)-1-(3,4-dichlorophenyl)ethyl]amino-2-hydroxypropyl](phenylmethyl)phosphinic acid (CGP55845; GABAB receptor antagonist), 4-ethylphenylamino-1,2-dimethyl-6-methylaminopyrimidinium chloride (ZD7288) (100 µM, h-channel antagonist) and 15 µM glycine, while MgSO_4_ was omitted. Spontaneous EPSC events were recorded from pyramidal cell-like neurons based on the morphology in voltage clamp configuration held at –77 mV including the liquid junction potential correction. The collected data were filtered at 1 kHz or 3 kHz and digitized at 10–20 kHz with the help of 1322A A/D board and with Clampex 10 program suite (Molecular Devices, Sunnyvale, CA). Series resistance was not compensated. Input resistance and series resistance values were monitored by injecting 5-mV steps. Then, 20 µM 6-nitro-7-sulfamoylbenzo (f)quinoxaline-2,3-dione (NBQX, Tocris Bioscience) was added to isolate the NMDA component of the sEPSCs. Finally, at the end of all experiments, 50 µM D-(-)-2-Amino-5-phosphonopentanoic acid (D-AP5, Tocris Bioscience) was added to verify that NMDA channel currents were blocked. The 20 largest events were collected from 2-minute long-continuous traces to assess the properties of the sEPSCs and the NMDA components in Table 1. The values of resting membrane potential, membrane time constant, and input resistance of pyramidal cells were measured in current clamp mode in some of these experiments.

### Behavioral Experiments

All mice were male and aged 10–16 weeks unless otherwise stated. Mice were weaned at postnatal three weeks and single housed for at least one week prior to behavioral testing. Housing was maintained on a 12 hr light/dark cycle (light 6 am to 6 pm) with food and water *ad libitum*. All behavioral experiments, except for the Y-maze tasks, were performed during the light phase by an investigator blind to genotype and treatment. All apparatuses were cleaned with 70% ethanol between animals during testing.

#### Novel object recognition tasks

Novel object recognition tasks were carried out as previously described with minor modifications [28]. The test apparatus was a rectangular plexiglass box with one large exploratory chamber (40×40×22 cm) and one adjacent smaller test chamber (40×20×22 cm), accessible through an aperture in the wall dividing the two chambers. Testing was completed in three stages: (1) First, animals were habituated to both test chambers for 10 min. (2) Immediately after habituation, animals were confined to the exploratory chamber for 3 min with two or five objects, distinctive only by shape, fixed to different locations within the chamber. (3) After a 2-min intertrial interval in the home cage or immediate transfer (<10 sec), animals were tested for object recognition in the test chamber. One familiar object from the exploration trial and one novel object were fixed within the test chamber and time spent investigating the two objects was video-recorded and scored manually. Object position and novelty were counterbalanced between genotypes.

#### Contextual fear conditioning task

Contextual fear conditioning task was conducted as previously described [63]–[64] with some modification. In brief, animals were placed into Context A, and after 3-min habituation, received one foot-shock (2 s, .8 mA) and were removed 1 min post-shock. No tones were used in this paradigm. Twenty four hr after training to foot-shock in Context A, mice were exposed to Context B for 3 min and then, after a 3-hr interval, were exposed to Context A for another 3 min. All fear conditioning tasks were scored with Med Associates FreezeView software.

#### Y-maze spontaneous alternation and spatial long-term memory tasks

The y-maze with three identical arms of transparent plexiglass (40×4.5×12 cm) 120° apart was placed in the center of a diffusely illuminated room (30 lx) with clues located in the periphery of the room to allow visual orientation.

The spontaneous alternation task was performed as previously described [10]. The spatial long term memory task was modified from prior methods [65]. In a 10-min exploration trial, one arm was blocked with plexiglass). After an interval of 180 min, the plexiglass divider was removed and the mouse was allowed to explore the three arms for 3 min. Both y-maze tasks were recorded through a camera mounted above the maze and evaluated by automatic video tracking (ANY-maze). All experiments were conducted during the dark phase (6∶00 p.m. to 9∶00 p.m.) to maximize exploratory behavior.

#### Open field, amphetamine-induced and mk-801-induced hyperlocomotion tests

Locomotor and exploratory activity in a novel open field was assessed as described previously [68] through photobeam breaks in a 40×40 cm VersaMax chamber (Accuscan Instruments). The chamber was placed in the center of a room (3×3 m) and was homogenously illuminated at 50 lx.

The drug-induced hyperlocomotion tests were conducted in the same VersaMax chambers at 50 lx, divided into four quadrants (20×20 cm); only two quadrants were occupied during testing. For amphetamine-induced hyperlocomotion test, animals received *i.p.* injection of *d*-amphetamine (2.5 mg/kg; Sigma-Aldrich) and were returned to the test chamber for another 120 min. Test mice were aged 16–20 weeks. A different cohort of animals received *i.p.* injection of MK-801 ((+)-MK-801 hydrogen maleate, 0.2 mg/kg in 0.9% saline, Sigma-Aldrich) and was returned to the test chamber for another 120 min.

#### Female urine sniffing test

The female urine sniffing test is a recently established model for assessing reward or pleasure-seeking behavior in rodents [67]. Briefly, following habituation a cotton tip applicator, the applicator was soaked with water and positioned in the home cage of the test animal for three minutes. Sniffing duration of the cotton tip was recorded manually during this time. 30–45 min following this control test, a new cotton tip applicator was soaked in female estrus urine for another three min home cage test.

#### Other behavioral tests

The auditory startle reflex test and its prepulse inhibition [68] test, saccharine preference test, elevated plus Maze task, nest building test, social recognition test, hot plate test, and rotarod test were conducted as previously described [10].

## Supporting Information

Figure S1 **Spatial distribution of Cre recombination in G35-3-Cre line at 12 weeks of age.** Visualized by X-Gal staining after crossing to a R26RLacZ reporter line. Parasagittal (left column) and coronal (right column) whole brain slices (50 µm thickness) after safranin-O counterstaining. Note that axonal fibers, but not somata, from cortical projection neurons were stained in dorsal striatum (a), nucleus accumbens (b), and olfactory tubercle (c). Axon fibers on corpus callosum (d), fimbria (e), fornix (f) were also stained. Non-somatic leaky staining of lacZ was also observed in some thalamic nucleus (lateral posterior thalamic nucleus, g) and lateral septum (h).
(DOCX)

Figure S2 **Assessment of basic behavioral function, pleasure-seeking, and anxiety-like behavior.** **A.** Mutant mice (black triangle, n = 8) showed normal adult weight and growth compared to the fGluN1 control mice (open square, n = 8)(two-way repeated measures ANOVA; F(1,56) = 0.044, *p* = 0.83). **B.** No difference was detected in the rotarod test between mutant (solid line, n = 4) and Flox (dotted line, n = 6) mice (two-way repeated measures ANOVA; F(1,72) = 0.004, *p* = 0.95). **C.** No difference was detected in the pain sensitivity test between mutant (n = 8) and Flox (n = 8) mice (two-tailed t-test; t(14) = 0.41, *p* = 0.34).
(DOCX)
